# Ghrelin as an Anti-Sepsis Peptide: Review

**DOI:** 10.3389/fimmu.2020.610363

**Published:** 2021-01-28

**Authors:** Nimisha Mathur, Syed F. Mehdi, Manasa Anipindi, Monowar Aziz, Sawleha A. Khan, Hema Kondakindi, Barbara Lowell, Ping Wang, Jesse Roth

**Affiliations:** ^1^ Laboratory of Diabetes, Obesity, and Other Metabolic Disorders, The Feinstein Institutes for Medical Research, Manhasset, NY, United States; ^2^ Center for Immunology and Inflammation, The Feinstein Institutes for Medical Research, Manhasset, NY, United States

**Keywords:** ghrelin, growth hormone, sepsis, cytokine storm, inflammation, anti-inflammation

## Abstract

Sepsis continues to produce widespread inflammation, illness, and death, prompting intensive research aimed at uncovering causes and therapies. In this article, we focus on ghrelin, an endogenous peptide with promise as a potent anti-inflammatory agent. Ghrelin was discovered, tracked, and isolated from stomach cells based on its ability to stimulate release of growth hormone. It also stimulates appetite and is shown to be anti-inflammatory in a wide range of tissues. The anti-inflammatory effects mediated by ghrelin are a result of both the stimulation of anti-inflammatory processes and an inhibition of pro-inflammatory forces. Anti-inflammatory processes are promoted in a broad range of tissues including the hypothalamus and vagus nerve as well as in a broad range of immune cells. Aged rodents have reduced levels of growth hormone (GH) and diminished immune responses; ghrelin administration boosts GH levels and immune response. The anti-inflammatory functions of ghrelin, well displayed in preclinical animal models of sepsis, are just being charted in patients, with expectations that ghrelin and growth hormone might improve outcomes in patients with sepsis.

## Introduction

Sepsis is a life-threatening organ dysfunction caused by a dysregulated host response to infection (1) which continues to be a leading cause of death in the United States. According to the Center for Disease Control, each year, at least 1.7 million adults develop sepsis in America and nearly 270,000 Americans die as a result of it. Sepsis causes an exaggerated production of pro-inflammatory cytokines by the activated immune-reactive cells that result in multiple organ failure leading to death (2). Despite the growing understanding of the pathophysiology of sepsis, progress has been slow in the therapeutic control of sepsis (3).

According to recent studies, peptide hormones of mammals like ghrelin, hCG, glucagon, glucagon-like peptide 1, and vasopressin have shown promising effects on sepsis, presumably based on their anti-inflammatory properties. In this review article, we focused on ghrelin and its mechanism of action on various organs in sepsis. Although the beneficial role of ghrelin in downregulating the expression of pro-inflammatory cytokines was reported in several studies (4, 5) the mechanism of action of ghrelin in sepsis has only been clarified within the last few years. Ghrelin was discovered to downregulate the expression of pro-inflammatory cytokines such as tumor necrosis factor α (TNF-α), interleukin (IL)-6, and IL-1β (4), while upregulating the expression of anti-inflammatory cytokines e.g., IL-10 and transforming growth factor (TGF) β (6).

**Figure 1:**
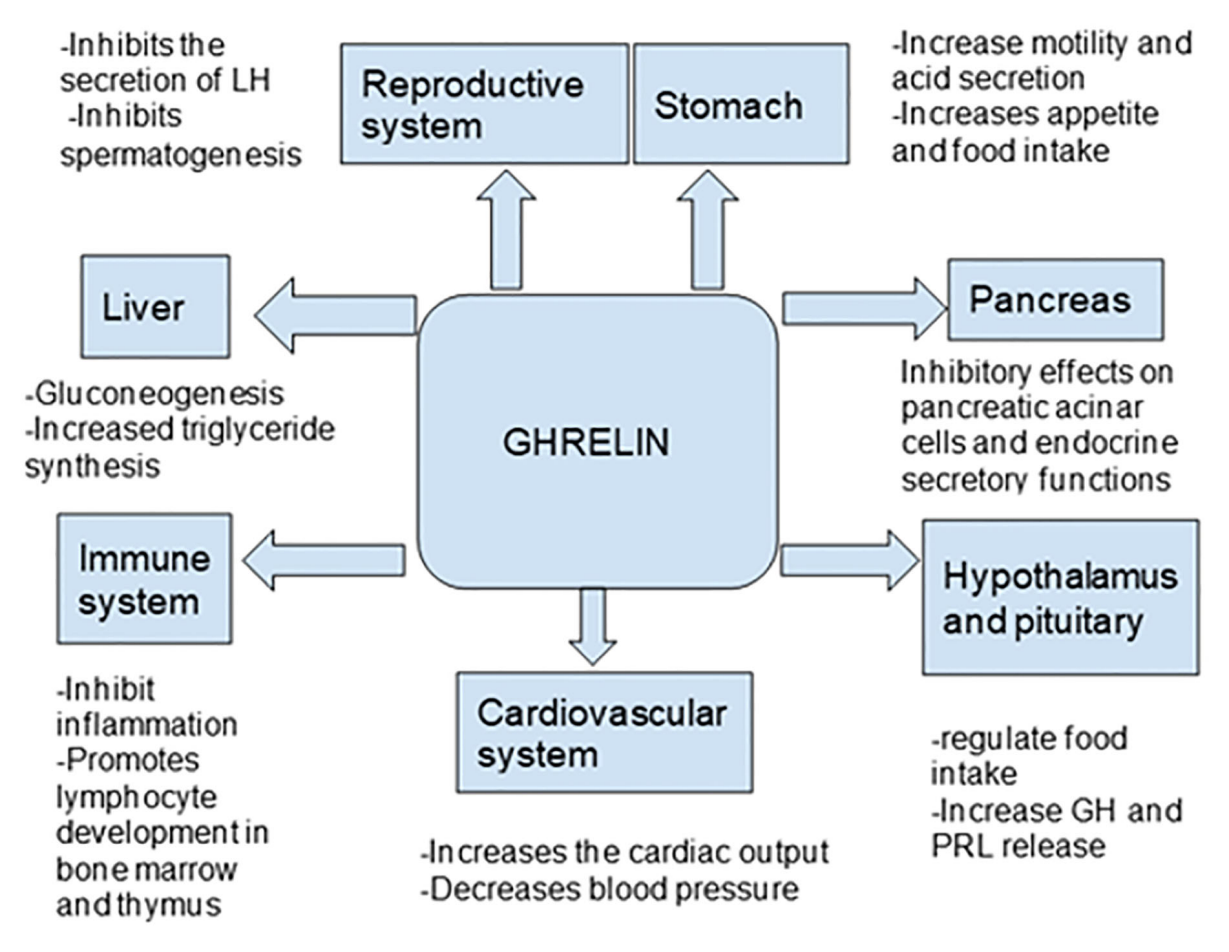
Effects of ghrelin on different organ systems (31–40).

Ghrelin, a peptide hormone, is widely recognized as a growth hormone secretagogue (7) and independently acts as an appetite stimulant. Besides these classic well-established roles of ghrelin, it has been shown to affect many organ systems including the central nervous system as well the cardiovascular, gastrointestinal, reproductive, and immune systems (**Tables 1** and **2**). Even after 20 years of its discovery, the role of ghrelin in regulating inflammation has not been well defined (7, 29–31) (**Figure 1**). In this review, we focus on the role of ghrelin in inflammation and its emerging usefulness in the treatment of sepsis.

**Table 1:** Physiology and biochemistry of ghrelin: (8–10).

Gene	Chromosome 3 (3q26.2)
Synthesis	Stomach ghrelin cells
Precursor	Preproghrelin, 117 amino acid peptides→ post-translational cleavage→ Proghrelin
Hormone	Ghrelin, 28 amino acid peptides
Receptor	GHSR (Growth hormone secretagogue receptor), G-protein coupled receptor
Main physiological role	Growth hormone release; Starvation, same as in calorie restriction in sepsis, causes to synthesize octonylated peptide ghrelin from proghrelin by Ghrelin O- acyltransferase (GOAT) which stimulates Growth hormone from pituitary cells to normalize blood glucose levels (10). Sepsis and prolonged illness can cause hyperglycemia. Given the impact of Ghrelin in controlling glucose homeostasis, the beneficial outcomes of Ghrelin treatment in sepsis could be mediated through maintaining normoglycemia in septic patients. Appetite stimulation; Anti-inflammation

**Table 2:** Anti-inflammatory role of ghrelin in animal and human studies.

Alzheimer’s disease (male Tg APPSwDI mice (11), male ICR mice (12)	Treatment with ghrelin reduced inflammation of the microglia. It also decreased tau phosphorylation *via* the PI3-K/Akt-GSK pathway in hippocampal neuron cultures. Ghrelin also decreased levels of amyloid-beta peptide (11–13).
Multiple sclerosis (Sprague Dawley rats) (14)	It decreases inflammatory brain infiltration, inhibits NF-κB, suppresses microglial aggregation around vessels in the rats (13, 14). Ghrelin also showed a growth promoting effect on neuronal cells by inducing modifications in the growth hormone secretagogue receptor type 1 (GHS-R1) expression (15).
Subarachnoid hemorrhage (Male Wistar albino rats)	Ghrelin reduced the proinflammatory mediators, prevented the reduction of endogenous antioxidants and preserved the endothelial integrity of cerebral arteries (16).
Acute lung injury in a traumatic brain injury (TBI mice model)	-Ghrelin reduced the activation of NF-κB, pro-inflammatory factors including IL-1β, IL-6, TNF-α, and IL-18, and the apoptosis related proteins like NLRP3, Caspase1-P20, and HMGB1 in the lung tissues (17) - It also reduced the peripheral macrophage invasion and reduced lung vascular permeability thereby improving lung function (17)
Intestinal injury due to traumatic brain injury, (Balb/c mice model)	Decreases intestinal permeability and TNF-α levels (18).
Chronic respiratory Diseases (human study)	-Ghrelin suppressed the airway inflammation by decreasing neutrophil accumulation, suppressing levels of IL-8 and TNF-α (19) (**Figure 2**) Ghrelin has also been shown to increase appetite and body weight in cachectic patients.
Atherosclerosis (mice model, lipid rich diet fed)	Ghrelin receptor agonists increased the cholesterol removal from macrophages and reduced the plaque formation in mice maintained on a high fat diet (20). Has an anti-inflammatory action in the regulation of atherosclerosis (20–23).
Gastric Ischemia reperfusion injury (Male Wistar rats (24, 25), Male Sprague Dawley rats (26)	1. Ghrelin reduced induction of cytokines, neutrophil infiltration, and production of reactive oxygen species (24). 2. The protective effect of ghrelin on gastric mucosa in mice is dependent on increased gastric blood flow, endogenous nitric oxide release, prostaglandin release, decreased TNF-α expression in inflammatory cells, and intact sensory nerves (25, 26).
Inflammatory bowel disease, Models -Sprague-Dawley rats of both sexes (19), Balb/c mice (6–8 weeks old) (27)	Ghrelin inhibits inflammation and protects the mucosa (19, 27)
Obesity (C57BL/6 mice model)	Ghrelin reduced mRNA expression of TNF-α, TGF-β, IL-1β, IL-6, and Collagen I (28)

## Ghrelin Synthesis

The *in vivo* growth hormone-releasing properties of ghrelin energized the path to its discovery, purification, and early naming but later recognition of a much richer menu of activities have subordinated the link to growth. Starting with rat stomach, Kojima et al. purified an endogenous ligand that activates GHSR-1A (growth hormone secretagogue receptor type 1A), a 28-amino acid peptide produced by the post-translational cleavage of preproghrelin (117 amino acids) (2, 3) After that cleavage, proghrelin is acylated by ghrelin O- acyltransferase (GOAT) at the third serine residue to produce the active hormone, acylated ghrelin (41–43). Ghrelin is released from the cells in the stomach that have compact electron dense granules. Most of these cells are in oxyn4tic mucosa of the fundus. Small numbers of cells are resident in pyloric glands of the stomach and the small intestine (7, 44–46). Most of the circulating ghrelin originates from cells of the gastrointestinal tract as well as the pancreas (46, 47).

## Ghrelin Receptors Are Involved in Anti-Inflammatory Activities of Ghrelin

The active ghrelin receptor, known widely as GHS-R1a, is a G-protein coupled receptor (GPR) (48) with seven transmembrane domains. GHS-R1a belongs to the sub-family of class A G-protein- coupled receptors that includes the motilin receptor, neurotensin-1 and –2 receptors and neuromedin -1 and -2 receptors (49). Growth hormone secretagogue receptor 1b, (GHS-R1b) is also referred to as a receptor but is involved in modulating the 1a receptor (7) to growth hormone secretagogue, a synthetic peptide agent or small molecule compound that releases growth hormone from the pituitary (50–52).

As ghrelin has a short half-life, owing to its de-acylation by esterase in the circulation, its synthetic analog, growth hormone-releasing peptide-2 (GHRP-2) was used to study GHS-R- (growth hormone secretagogue receptor) mediated effects. Anti-inflammatory effects of GHRP-2 occur by direct stimulation of immune cells, mediated by ghrelin receptors. GHRP-2 inhibited lipopolysaccharide-induced IL-6 release from peritoneal macrophages *in vitro* from arthritic rats. GHRP-2 also decreased production of ACTH and corticosterone, which are elevated in arthritic rats. It was also shown that human T- lymphocytes and monocytes expressed GHS- R, and ghrelin inhibits production of cytokines IL-1β, IL-6, and TNF-α *via* GHS-R (49).

## Distribution and Action of Ghrelin Receptors

**Figure 2:**
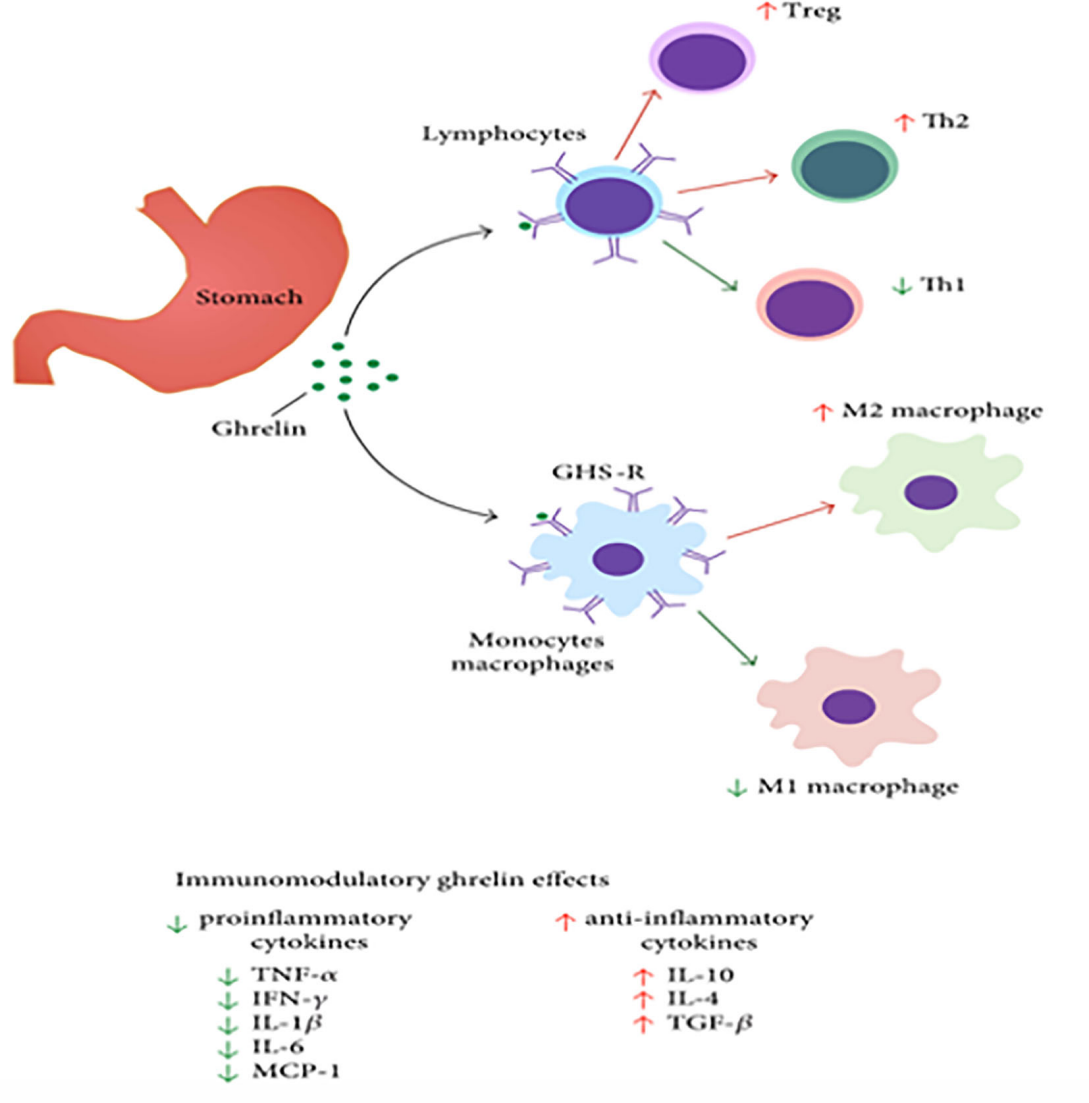
Ghrelin’s impact on immune cells, like macrophages, lymphocytes, and non-immune cells like epithelial or endothelial cells. [Modified from (55, 56)].

The GHS-R-1a receptors are expressed in many organs including lung (especially alveolar macrophages), kidney, heart, intestine, liver, and adipose tissue (2, 3, 29). Other prominent sites include the arcuate and ventromedial nuclei of the hypothalamus (53). They are also distributed on immune cells including monocytes, monocyte-derived dendritic cells, and T cells (53, 54). Typically, on activation of these immune cells, the G protein coupled GHS-R1a receptor expression is significantly increased, leading to increased mobilization of calcium as well as cytoskeletal changes (55) (**Figure 2**). While the calcium mobilization *via* a G-protein coupled receptor is common, the extent of calcium release linked to ghrelin receptors is very prominent, analogous to the large amounts released by some of the very potent T-cell chemokine ligands (55), When T cells are activated, the ghrelin receptor expression is significantly increased which leads to increased influx of calcium. Also, ghrelin decreases the levels of IL-1 β and IL-6 from LPS treated monocytes. These effects are reversed when treated with ghrelin receptor antagonists (55). Overall, these findings suggest the important role of ghrelin and ghrelin receptors in inflammation.

## Anti-Inflammatory Actions of Ghrelin in Experimental Mouse Models

The anti-inflammatory role of ghrelin has been established widely in diseases affecting many systems including the central nervous system, as well as immune, digestive, respiratory, skeletal, metabolic, and endocrine systems. Treatment with ghrelin decreases inflammation, thereby reducing the disease severity in multiple disorders including sepsis, inflammatory bowel disease, arthritis (57), pancreatitis, obesity (58–61), diabetic nephropathy (49, 62–65), cachexia, experimental autoimmune encephalomyelitis (66), and several mouse models of autoimmune chronic inflammatory states (13).

## Immune System

One major role of ghrelin is to decrease inflammation from sepsis (67). The anti-inflammatory role of ghrelin has been studied in mouse models of sepsis. Post-treatment with a bolus intravenous injection of ghrelin into mice with sepsis significantly reduced the serum levels of TNF-α and IL-6 (4).

## Neuroprotection

Ghrelin promotes neuroprotective effects *via* its anti-inflammatory properties. In rats, it reduces the brain damage induced by subarachnoid hemorrhage by decreasing the inflammation and preserving the endothelial integrity of cerebral arteries (16). Ghrelin receptor agonists also decrease beta-amyloid peptide and inflammation in the microglia of murine models of Alzheimer’s disease (11, 12). It also delays and diminishes the symptoms in the experimental autoimmune encephalitis rat model of multiple sclerosis (13, 14).

## Respiratory Illnesses

In mice suffering from a chronic respiratory disease model, intravenous ghrelin decreases the airway inflammation (13). Similar benefits of ghrelin have also been observed in mouse models with acute lung damage associated with traumatic brain injury (17), and pancreatitis (68), as well as administration of bleomycin (69), oleic acid (70), and monocrotaline (71). In patients with asthma, ghrelin levels inversely correlated with disease severity (72).

## Vascular Diseases

In mouse models, ghrelin reduced inflammatory responses associated with ischemia-reperfusion injury including ulceration, tissue congestion, and neutrophil infiltration (24). It also was associated with a significant increase in gastric blood flow and reduction in gastric erosions (25, 26, 73).

## Digestive System

Traumatic brain injury alone often results in dysfunction of multiple other organ systems. The intestines become inflamed with increased permeability and translocation of microbes. Ghrelin administration mitigates these alterations (18). In mice with colitis induced by trinitrobenzene sulfonic acid (TNBS), the production of chemo-attractants, chemokines, chemokine receptors and other pro-inflammatory mediators is tempered by ghrelin and IL-10, another anti-inflammatory cytokine (27). Ghrelin treatment is also associated with a remarkable increase in colonic blood flow compared to control animals.

In rats, when sensory nerves in the colonic mucosa are inactivated by capsaicin, the healing and vasodilatory properties of ghrelin are reduced, suggesting a role of sensory nerves in the healing promoted by ghrelin.

These experiments support the involvement of nitric oxide, prostaglandins, and sensory nerves in the protection of colonic mucosa by ghrelin. It is to be noted that in ulcerative colitis patients, the mRNA expression of ghrelin is significantly upregulated in inflamed colonic mucosa (74).

## Metabolic Diseases

Des-acyl Ghrelin has been shown to prevent the development of pre-diabetes in a mouse model fed with a high fat diet, showing its potential therapeutic role in type 2 diabetes (75). It also reduced inflammation in mouse models of obesity (20–23, 28, 58–61, 76, 77).

## Anti-Fibrotic Properties

Ghrelin has anti-inflammatory properties that are protective, reducing fibrosis in heart (myocardial infarction, chronic hypertension, doxorubicin induced cardiac fibrosis), lungs (idiopathic pulmonary fibrosis, diffuse fibrosing alveolitis, diffuse interstitial fibrosis), liver (viral infections, alcohol abuse, non-alcoholic fatty liver disease), kidneys (renal interstitial fibrosis), and skin (systemic sclerosis) (78).

## Mechanisms Involved in the Protective Role of Ghrelin in Inflammation

### Inhibition of Pro-Inflammatory Cytokine Production

Ghrelin inhibits the production of pro-inflammatory cytokines from monocytes, T-cells, and macrophages (55). It also inhibits leptin-induced pro-inflammatory cytokine expression including IL-1β, IL-6, and TNF-α in human T lymphocytes and monocytes (55, 79).

### Ghrelin Inhibits HMGB1 (High Mobility Group Box 1) Protein

HMGB1 is a nuclear protein encoded by the HMGB-1 gene. It is also transformed into a cytokine secreted by many immune cells including monocytes, macrophages, and dendritic cells (80). HMGB1 is also involved in stimulating the release of pro-inflammatory cytokines, including TNF- α, IL-1, IL-6, and IL-8, thereby amplifying inflammation (81). In a mouse model, HMGB1 was released by cultured macrophages 8 h after stimulation with endotoxin, TNF- α, or IL-1. Ghrelin treatment of macrophages inhibits the translocation of HMGB1 from nucleus to cytoplasmic compartment, thereby abrogating its secretion from activated (LPS-stimulated) macrophages. Ghrelin also reduces bacterial load in sepsis, which also indirectly contributes to less HMGB1 secretion (55, 82).

The delayed secretion of ghrelin by macrophages, stimulated by LPS, IL-1, and TNF- α may be part of a homeostatic checkpoint to prevent an all-out inflammatory response (83).

### Ghrelin Inhibits NF-kB and Other Genes Involved in Inflammation

In sepsis, when the host immune system is activated by pathogen-associated molecular patterns (PAMPs) or damage-associated molecular patterns (DAMPs), a cascade of intracellular signal transduction pathways are stimulated, which lead to the activation of downstream transcription factor NF-κB and mitogen-activated protein kinase (MAPK). These activated signaling cascades enhance the expression of pro-inflammatory cytokines including IL-1β, TNF-α, IL-6, and IL-12 (84). NF-κB is involved in the pathogenesis of acute and chronic inflammatory diseases including sepsis. NF-κB/Rel proteins include NF-κB2 p52/p100, NF-κB1 p50/p105, c-Rel, RelA/p65, and RelB. These proteins function as dimeric transcription factors that regulate the expression of genes relevant to a wide range of biological processes such as innate and adaptive immunity as well as inflammation. While inactive, NF-κB/Rel proteins are bound and inhibited by IκB proteins. The PAMPs, DAMPs, pro-inflammatory cytokines, and antigen receptors activate an IKK complex (IKKβ, IKKα, and NEMO) that phosphorylates IκB proteins. Phosphorylation of IκB leads to its ubiquitination and proteasomal degradation, releasing NF-κB/Rel complexes. Active NF-κB/Rel complexes translocate to the nucleus and induce target gene expression (84, 85). Intriguingly, several studies demonstrated that ghrelin inhibits the translocation of LPS-induced NF-κB (p65) into the nucleus in murine macrophages, which explains the suppressive effects of ghrelin on pro-inflammatory cytokine production by the macrophages after LPS-stimulation (6, 86).

The MAPK signaling pathways play an important role in increasing inflammation. Mitogen-activated protein kinase phosphatase-1(MKP-1) provides a negative feedback signal to decrease the activity of MAPKs (87). Studies have shown that the anti-inflammatory effect of ghrelin is due to increased expression of MKP-1, further decreasing the inflammation (61, 88).

### Ghrelin Increases the Release of Anti-Inflammatory Cytokines

IL-10 is a cytokine that decreases the production of pro-inflammatory cytokines including IL-1α and -1β, IL-6, IL-12, IL-18, and TNF-α (85). In some studies, it has been found that ghrelin increases the production of IL-10 by enhancing the p38 MAPK (mitogen-activated protein kinase) phosphorylation *via* the GHSR (6, 27, 60), which inhibits inflammation by controlling the production of pro-inflammatory cytokines.

## Role of Ghrelin in the Nervous System

The autonomic nervous system plays an important role in inflammation. The activation of the sympathetic nerves stimulates the release of pro-inflammatory cytokines such as TNF-α (89, 90). In sepsis, organ failure is promoted by stimulation of the sympathetic nervous system (91–93). Ghrelin protects experimental animals from sepsis-induced organ failure by suppressing the sympathetic nervous system (94–97).

The activation of the parasympathetic nervous system leads to reduced systemic levels of pro-inflammatory cytokines including TNF-α (90). Acetylcholine inhibits the release of IL-1α, IL-1β, and IL-6 cytokines.

Stimulation of vagus nerve attenuates LPS-induced endotoxic shock. It has also been shown that ghrelin can stimulate the vagus nerve (4, 90). Ghrelin administration to vagotomized polymicrobial septic mice did not reduce the levels of TNF-α, IL-6, AST, ALT, and lactate in contrast to control groups (4). Intact vagus nerve protects the mice from sepsis-induced inflammation and organ injury. This implies that ghrelin mediates its anti-inflammatory properties and protects the experimental animals from organ failure by stimulating the cholinergic anti-inflammatory pathway (4, 98).

## Functions of Ghrelin on T Cells

During sepsis, although the initial increase in anti-inflammatory cytokines is important, prolonged increases can lead to immunosuppressive conditions resulting in secondary infections. Successful outcomes require a balance between pro- and anti-inflammatory agents. In sepsis there is a reduction in CD4 T cells, significantly tempering the immune response to infections (99). Treatment with ghrelin rescued the CD4+ population from apoptosis (5, 100). Therefore, ghrelin exerts a protective effect by increasing the proliferation of CD4 T cells and decreasing their loss to apoptosis (101).

## Interaction of Ghrelin With Melanocortin System

One group of investigators have suggested that interaction with elements of the melanocortin system is another possible mechanism by which ghrelin exerts its anti-inflammatory properties. In sepsis and critically ill patients, dysfunction of the hypothalamic-pituitary-adrenal axis occurs leading to corticosteroid insufficiency. Administration of ghrelin stimulates the release of CRH, ACTH and corticosterone by acting on HPA axis (5, 32, 102) (**Figure 1**). This pathway likely mediates ghrelin effects against inflammation.

## Inhibition of Th17 Cells

Th 17 cells are members of pro-inflammatory T-helper cells and therefore are activated during multiple pro-inflammatory events. Th 17 cells increase the production of IL-17. Mice with knockdown of ghrelin showed significant elevation of IL-17 in T-cells (103). Ghrelin inhibits differentiation of Th17 cells both *in vitro* and *in vivo*, thereby exerting anti-inflammatory function. In mice with experimental autoimmune encephalitis, treatment with ghrelin prevented infiltration of Th17 cells and decreased brain damage (104).

## Autophagy

Autophagy is another important mechanism by which ghrelin can protect organs from injury. Autophagy means eating of self in Greek and plays an important role in clearing damaged organelles such as peroxisomes, endoplasmic reticulum, and mitochondria (105). In many diseases, ghrelin protects neurons, intestinal epithelial cells, vascular smooth muscle cells, heart cells, and hepatocytes from cellular damage by inducing autophagy (106).

## Antimicrobial Properties of Ghrelin

Multiple studies showed the microbicidal actions of ghrelin. Anti-microbial properties of ghrelin—both acylated and des-acylated forms of ghrelin possess potent killing properties against microbes, especially gram-negative bacteria. Recall that it is the acylated ghrelin that is the bioactive entity on mammalian entities while the des-acylated versions are not active on mammalian molecular entities.

Des-acyl ghrelin (DAG) was originally thought to be an inert form of acyl ghrelin (AG). However, recent data suggest that DAG can antagonize some aspects of AG function in addition to having GHSR-independent effects.

It is postulated that DAG activates its own receptor and also can interact with AG at this receptor. DAG can be a functional inhibitor of ghrelin and suppress ghrelin levels in humans. Therefore, DAG or DAG analogs can be good candidates for future treatment of metabolic disorders in which antagonism of AG actions could be beneficial like obesity, diabetes, and Prader- Willi syndrome (107).

Like many cationic antimicrobial proteins, ghrelin’s positive charge was able to counteract the negative charge of bacterial cell walls (108). Ghrelin also reduces the bacterial load in the peritoneal fluid of cecal ligation and puncture (CLP)-induced septic mice and demonstrated bactericidal effects on E. coli (109).

## Role of Ghrelin in Sepsis

### Effect of Ghrelin on Cell Studies

**Figure 3:**
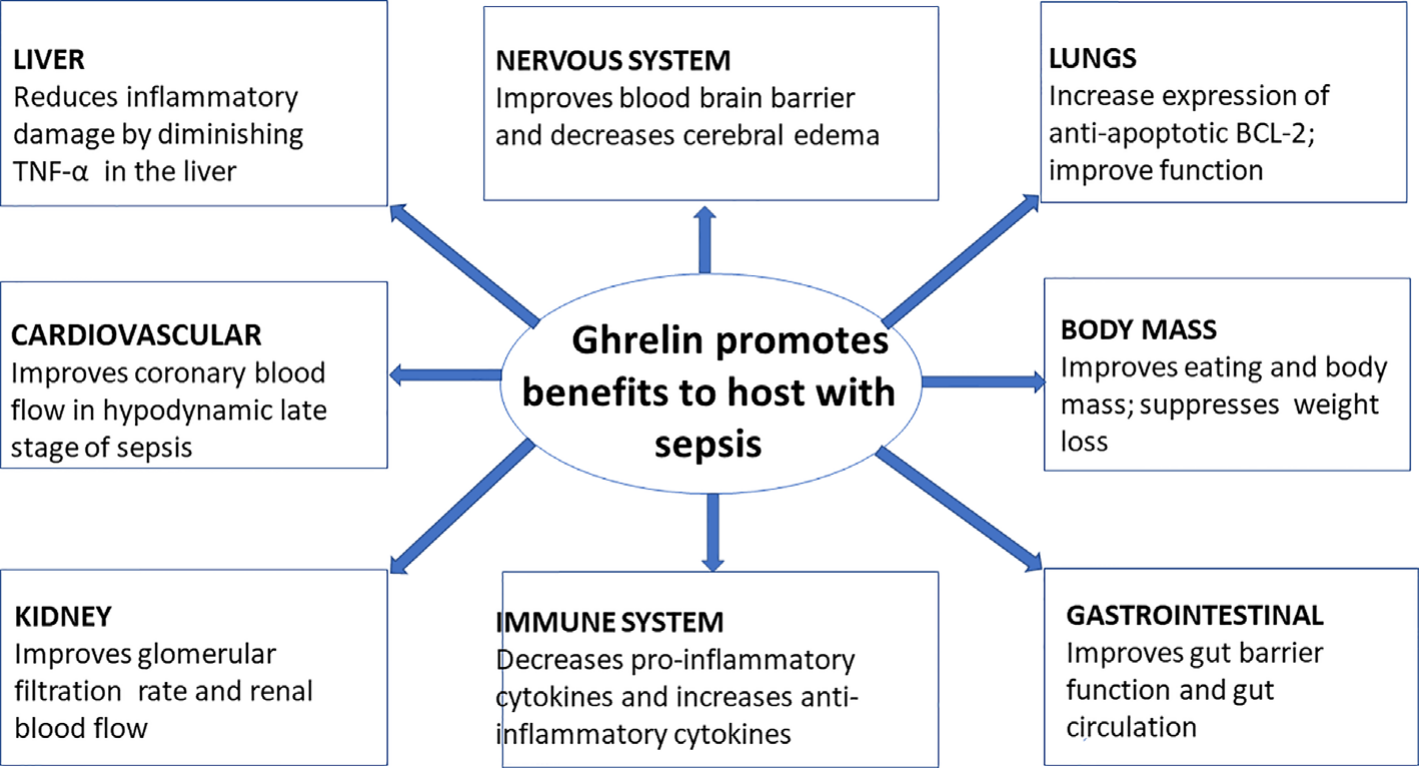
Ghrelin and its effect on various organs during sepsis (110–126).

In vitro, several studies demonstrated the immunoregulatory role of ghrelin (**Figure 3**). Treatment with ghrelin inhibited the release of pro-inflammatory cytokines IL-1β, IL-6, and TNF-α from human monocytes, T cells, and peripheral blood mononuclear cells (PBMCs) (55, 66). Ghrelin also decreased an adipocytokine leptin-induced IL-1β, IL-6, and TNF-α protein and gene expression by cultured T cells (55, 127, 128). Also, ghrelin treatment decreased the TNF-α-induced release of IL-8 and MCP-1 and the activation of NF-κB in human umbilical vein endothelial cells (HUVEC) (60). Likewise, ghrelin also decreased the mRNA expression of TNF-α in lymphoid cell lines of children with autism (129). Ghrelin impedes the release of pro-inflammatory cytokines from LPS-stimulated microglial cells (66). It also prevents the release of IL-6 from LPS-stimulated dopaminergic neurons (130). Ghrelin administration reduced LPS-induced expression of proinflammatory cytokines, and inducible NOS in alveolar macrophages. It also reduced LPS-induced NF-κB translocation in alveolar macrophages (131).

### Effect of Ghrelin in Animal Studies

Several *in vivo* studies highlighted the anti-inflammatory role of ghrelin in sepsis. In mice with LPS-induced endotoxemia, ghrelin administration produced significant inhibition of release of TNFα, IL-6, and IL-1α (80). In another study in mice, intravenous administration of ghrelin reduced pro-inflammatory cytokine (TNFα, IL-8, and MCP-1) production induced by LPS (60). LPS administration in mice increased levels of TNFα and IL-6; ghrelin reduced the cytokine levels significantly (132). After CLP operation in rats, ghrelin administration led to decreased levels of TNFα and IL-6 (4). Ghrelin decreased both early and late mediators of inflammation. Chorny et al. showed that ghrelin, even 12–24 h after CLP, improved mortality in mice and significantly reduced both clinical parameters and histopathological scores of sepsis, thus proving its effects on late mediators of inflammation (57).

### Effect of Ghrelin on Nervous System in Sepsis

As discussed earlier, the autonomic nervous system plays an important role in inflammation. While the sympathetic nervous system is pro-inflammatory, the parasympathetic nervous system is anti-inflammatory (96). Ghrelin has an inhibitory effect on the sympathetic nervous system and a stimulatory effect on the parasympathetic nervous system. In sepsis, cytokines released by the liver macrophages (also known as Kupffer cells) cross the blood brain barrier and stimulate the hypothalamus and rostral ventrolateral medulla to increase the central sympathetic outflow, causing significant release of catecholamines into the blood which causes further damage to the liver (96, 133). These catecholamines act on adrenergic receptors in the liver and stimulate the release of unusually high amounts of TNF-α (134). Ghrelin has been shown to inhibit sympathetic neurons both centrally and peripherally. It has also been shown to stimulate the vagus nerve (96, 133). Ghrelin decreased cerebral edema and improved blood brain barrier integrity by decreasing the inflammatory response in the brain of septic mice. It also protected neuronal tissue from oxidative stress and apoptotic damage (135).

### Ghrelin Effects on Cognition in Sepsis

Ghrelin administration in a mouse model induced angiogenesis and neurogenesis in the hippocampus (136). After CLP in rats, ghrelin significantly lowered pro-inflammatory cytokines and inhibited caspase-3 in the hippocampus. It also reduced the density of apoptotic neurons and improved cognition in ghrelin-treated rats compared to the control group (137). In addition, ghrelin diminished cognitive impairment associated with septic encephalopathy (138). The expression of ghrelin receptors are reduced with increased age. Therefore, the ghrelin treatment in aged animals is refractory. However, combined treatment with ghrelin and growth hormone overcomes ghrelin unresponsiveness and provides protective role against sepsis. Ghrelin with growth hormone administration after CLP resulted in c-fos expression, a marker of neuronal activation in the brain (139).

### Effects of Ghrelin on Respiratory System in Sepsis

Acute respiratory distress syndrome (ARDS) is another complication associated with sepsis (140). Administration of ghrelin improved pulmonary function and mortality in mice, associated with inhibition of NF-κB and reduction of pro-inflammatory cytokines in the lungs (139). In a study done with a synthetic agonist of the ghrelin receptor, LPS-induced lung injury in mice was ameliorated by inhibiting the NF-κB signaling pathway, which eventually decreased the pro-inflammatory cytokine production (141). In mouse models, when ARDS was induced with CLP, ghrelin administration inhibited apoptosis of alveolar macrophages (142), protected the integrity of the microvascular barrier and decreased the neutrophil activity in the alveoli (142–144). It has also been found that ghrelin improves outcome in many other lung diseases like pneumonia, pulmonary edema, emphysema, and cystic fibrosis (110, 111). Furthermore, treatment with ghrelin showed improvement in lung injury score, by decreasing pulmonary edema, hemorrhage, congestion, and inflammatory cell infiltration in septic mice (132). Ghrelin treatment also reduced the expression of pro-apoptotic BAX and pro-inflammatory TNF-α but increased the expression of anti-apoptotic BCL-2 in LPS-treated mice (112). In this study, tissue superoxide dismutase levels have also been found to be increased with ghrelin administration. In this study, these mechanisms helped to decrease lung injury in mice due to sepsis (112). Another study also reported the beneficial effect of ghrelin on acute lung injury from sepsis by inhibiting NOD2/NF-κB signaling pathway (86). In this septic mouse model, ghrelin protected the lung tissue from sepsis, by inhibiting neutrophil infiltration and decreasing pro-inflammatory cytokine expression (86). In conclusion, ghrelin improved pulmonary function in mice by decreasing pro-inflammatory cytokines and increasing the expression of anti-apoptotic BCL-2.

### Effect of Ghrelin on Cardiovascular System in Sepsis

In sepsis, another important target is the cardiovascular system. The heart responds to the sepsis by increasing the blood flow initially and then followed by a hypodynamic phase (145). It was suggested by Wang et al. that decreased ghrelin levels in late stages of sepsis might be responsible for low volume states (146). During the hypodynamic phase, endothelin-1 is upregulated which leads to increased vascular resistance leading to decreased organ perfusion (113). Treatment with ghrelin after CLP in mice showed improved cardiac output and stroke volume and thereby improved the organ blood flow in hypodynamic late stage of sepsis. This action of ghrelin is mediated by downregulating endothelin 1 (114). In another study, ghrelin treatment after CLP improved ventricular peak systolic pressure and cardiac contractility by decreasing cytokines like TNF-α and by ghrelin’s direct effects on ventricular contraction (115, 116).

### Effect of Ghrelin on Gastrointestinal System in Sepsis

While TNF-α and IL-1 are released early by the macrophages in inflammation, HMGB-1 is a late mediator in sepsis (83). HMGB-1 contributes to the increases in gut permeability in sepsis including increase in translocation of bacteria (147, 148). In rats, intravenous injection of ghrelin after CLP showed significant reduction in gut permeability and bacterial translocation. This experiment highlighted the important role of ghrelin on gut barrier in sepsis.

Recently, ghrelin has been shown to increase the gastric blood flow, decrease the expression of pro-apoptotic factors like Bax, cleaved caspase-3, and increase the expression of Bcl-2 (149). In a CLP-induced septic mouse model, administration of ghrelin decreased weight loss and intestinal mucosal damage by reducing inflammatory responses. It also improved the function of intestinal epithelial cells of rats (117). In another rat model, treatment with ghrelin after CLP increased the expression of autophagy-associated proteins. This further supports that ghrelin protects intestinal mucosa from sepsis injury (117–121).

### Effect of Ghrelin on Liver

Ghrelin also protects the liver from organ failure (91–93). Treatment of LPS-induced septic mice with growth hormone releasing peptide-2 (GHRP-2), a ghrelin receptor agonist, prevented liver damage. While LPS caused significant elevation of liver enzymes and nitrites/nitrates, ghrelin receptor agonist administration inhibited these increases. In LPS-treated mice, this agonist also increased Insulin-like growth factor-1 (IGF-1) concentration in the serum and expression in hepatocytes (150). IGF-1 plays a significant role in preventing liver damage. Ghrelin receptor agonists also decreased TNF-α expression in the liver, thereby decreasing inflammatory liver damage (122). Ghrelin reduced the hepatic concentration of LPS-induced TNF-α, IL-1β, and IL-6.

### Effect of Ghrelin on Musculoskeletal System in Sepsis

Ghrelin showed significant effects on muscle metabolism. Ghrelin administration to LPS-induced septic rats showed less muscle damage when compared to control groups (123). In another study, prolonged peritonitis was induced with zymosan to measure the influence of ghrelin on muscle metabolism in critical illness. This peritonitis caused significant weight loss and reduced food intake in mice. Continuous administration of ghrelin to these mice improved food intake and body mass, but did not increase muscle mass or function (124).

### Ghrelin Effects on Kidneys in Sepsis

TNF-α is a major cytokine involved in the sepsis-related kidney injury (125, 126). Ghrelin administration after CLP decreased renal cytokines IL-1β, TNF-α, IL-6, endothelin, renal inducible nitric oxide synthase (iNOS), and serum NO (nitric oxide). Elevated levels of renal iNOS and NO are responsible for systemic arterial vasodilation which causes renal vasoconstriction leading to acute kidney injury. Ghrelin improved glomerular filtration rate (GFR) and renal blood flow (RBF) in the treatment group when compared to the control group (126). In another study, treatment with ghrelin in CLP-induced septic mice improved inflammatory cytokine levels, kidney function, and arterial blood pressure (151).

### Synergistic Effects of Growth Hormone and Ghrelin

Most experiments done on sepsis studied only a single hormone, but this study combined treatment with two different hormones. Wang et al. showed that combined treatment of ghrelin and growth hormone to aged mice after CLP resulted in improvement in the serum levels of IL-6, TNF-α, lactate, lactate dehydrogenase, creatinine, and blood urea nitrogen. It also showed decreased apoptosis of CD4 T cells and expression of inhibitory co-receptors of T cells (99, 141). Growth hormone was added to ghrelin to increase sensitivity of ghrelin receptors (152) in older mice as the expression of ghrelin receptors in aged septic mice is decreased. Addition of growth hormone to ghrelin in septic rats protected lungs, liver, kidneys, heart, and brain from inflammatory damage (141). Combined treatment also provided enhanced immune response from splenocytes to stimulation from LPS and anti-CD3/anti-CD28 antibodies. It also repairs HLA-DR expression, a major histocompatibility complex required by antigen presenting cells in aged septic mice (153). Recent studies demonstrated that growth hormone and ghrelin given together caused inhibition of TGF-β *via* vagus nerve stimulation, thereby maintaining the immune response in septic mice (67).

### Ghrelin Improves Radiation-Exposed Septic Mice

Administration of ghrelin also decreases organ injury and mortality in rats suffering from sepsis after radiation exposure. Exposure to radiation causes gut barrier disruption, which leads to the translocation of intestinal flora to the systemic circulation, leading to inflammation and remote organ injury (154). First, rats were given radiation of 5-Gy followed 48 h later by polymicrobial sepsis introduced *via* CLP. Two doses of ghrelin were given immediately after radiation exposure and with CLP. Ghrelin decreased both the attendant weight loss and mortality (99, 154).

### Ghrelin in Clinical Studies

Despite the success of ghrelin in improving outcomes for septic rodents, studies in humans are surprisingly sparse. When LPS was administered to healthy volunteers, ghrelin was one of the first hormones to be elevated (155). In neonates with infection (156), ghrelin levels were elevated. Patients with postoperative intra-abdominal sepsis showed a significant increase in ghrelin levels (157). In another study, ghrelin levels were elevated in sepsis patients and levels were inversely related to length of stay in ICU and to SOFA score (134). In another study, lower ghrelin levels were noted in ICU patients, compared to healthy controls (8, 133). In patients admitted to ICU for sepsis, ghrelin levels correlated inversely with the length of mechanical ventilation and length of ICU stay (9). In another study, higher serum ghrelin levels in sepsis patients at the time of admission correlated positively with their survival (10). Patients who did not need mechanical ventilation had higher ghrelin levels compared to patients who needed mechanical ventilation. No difference was noted between the concentrations of ghrelin in sepsis versus non-sepsis patients in critically ill ICU patients (10). Overall, *in vivo* ghrelin levels correlated with better clinical outcomes, but bias favoring positive results has not been excluded.

## Concluding Remarks

Ghrelin was discovered because of its ability to stimulate growth hormone release. Cells in and around the mucosa of the stomach release ghrelin, a 28-amino acid peptide that promotes growth hormone release from the pituitary and stimulates the appetite.

While most of the ghrelin-producing cells are in the oxyntic mucosa of the stomach, they are found in smaller amounts in and around the fundus of the stomach. Receptors for ghrelin are widely distributed in major organs throughout the body including the CNS and elements of the immune system. The major role of ghrelin may be to act widely to down-regulate inflammatory elements and to promote anti-inflammatory pathways widely throughout the body.

Sepsis occurs from direct microbial infection as well as sterile inflammation caused by ischemia reperfusion injury, post-surgery, and exposure to chemical substances. Moreover, organ transplanted patients are susceptible to infection because of their compromised immune systems. Ghrelin, its agonists, and growth hormone promise to shed light on the therapeutic avenues for the treatment of inflammatory diseases to mitigate organ dysfunction, septic shock, and reduce mortality.

### Current Status and Future Direction

Sepsis, characterized by widespread inflammation with organ dysfunction, continues to be a major cause of illness, disability, and death at all ages. Despite the recruitment of talented professionals and enhanced funding, management of sepsis has advanced slowly. Ghrelin, an anti-inflammatory peptide isolated from cells of the stomach lining, is a major candidate for therapy because of its power to suppress pro-inflammatory processes and enhance anti-inflammatory activities widely throughout the body. In animal studies, ghrelin has beneficial effects widely; effects on the CNS and the immune system are the most dramatic. Surprisingly, studies in unison with other candidate therapies and studies in humans are sparse.

## Author Contributions

All authors contributed to the article and approved the submitted version.

## Conflict of Interest

The authors declare that the research was conducted in the absence of any commercial or financial relationships that could be construed as a potential conflict of interest.
